# Exploring the Regulatory Mechanism of Total Alkaloids from *Portulaca oleracea* L. in UC Treatment Based on Network Pharmacology

**DOI:** 10.3390/ijms26146978

**Published:** 2025-07-20

**Authors:** Tianci Zhang, Linran Gao, Qianying Wang, Jiahui Zheng, Xinyu Wang, Meng Jiang, Kaixin Wu, Jinxia Ai

**Affiliations:** 1 School of Medical Technical, Beihua University, Jilin 132013, China; ajx@beihua.edu.cn (T.Z.); glr@beihua.edu.cn (L.G.); wqy@beihua.edu.cn (Q.W.); zjh@beihua.edu.cn (J.Z.); wxy@beihua.edu.cn (X.W.); jm@beihua.edu.cn (M.J.); wkx@beihua.edu.cn (K.W.); 2 School of Laboratory Medicine, Beihua University, Jilin 132013, China

**Keywords:** *Portulaca oleracea* total alkaloids, UC, network pharmacology, TLR4, NF-κB

## Abstract

This study aimed to investigate the potential mechanisms of action of total alkaloids from *Portulaca oleracea* L. (POL) on ulcerative colitis (UC) using a network pharmacology approach. Network pharmacology analysis identified two bioactive alkaloids within POL as primary anti-UC constituents, targeting 16 core therapeutic proteins and 113 UC-associated signaling pathways. To further explore the therapeutic effects, in vitro cell assays and in vivo animal experiments were conducted. In vitro, high concentrations of *Portulaca oleracea* total alkaloids (POAs) demonstrated dose-dependent cytotoxicity, significantly reducing Caco-2 cell viability and impairing migration. In a murine model of UC, disease induction led to substantial weight loss, elevated disease activity index (DAI) scores, colon shortening, and severe colonic tissue damage compared to controls. Furthermore, the UC group displayed significantly upregulated serum levels of pro-inflammatory cytokines, TNF-α and IL-1β, as well as increased protein and mRNA expression of TLR4 and NF-κB in colon tissues. Crucially, POAs treatment effectively ameliorated UC symptoms in mice, significantly reducing DAI scores, mitigating colon shortening, and markedly suppressing TLR4/NF-κB pathway activation. These findings strongly suggest that the therapeutic effects of POAs in UC are, at least in part, mediated by the inhibition of the TLR4/NF-κB signaling pathway, leading to a reduction in colonic inflammation.

## 1. Introduction

UC is a common chronic, recurrent immune-mediated inflammatory bowel disease (IBD) marked by chronic mucosal inflammation in the colon. Patients suffer from recurrent symptoms such as abdominal pain, bloody diarrhea, and fatigue, which severely compromise their quality of life and mental health, often leading to anxiety and depression [1,2]. The pathogenesis of UC involves multiple factors, including genetic predisposition, mucosal barrier dysfunction, intestinal microbiota disorders, abnormal immune regulation, and environmental triggers [3,4,5]. Current treatment methods, including aminosalicylates, corticosteroids, and immunosuppressants have limitations such as single targets, adverse reactions, poor tolerability, and high recurrence rates. This highlights the need for safer and more effective treatments [6].

*Portulaca oleracea* L. (purslane), a traditional Chinese medicinal herb, has been empirically used to manage intestinal disorders such as dysentery and chronic diarrhea. Modern pharmacological studies reveal that its bioactive compounds exhibit broad anti-inflammatory and antimicrobial properties [7,8]. The progression of ulcerative colitis is driven by various factors and is controlled by the TLR4/NF-κB signaling pathway [9,10,11]. Pathological studies have confirmed that this pathway is over-activated during intestinal injury, which is associated with the progression of ulcerative colitis [12]. The precise regulation mechanism of purslane in ulcerative colitis is still unclear. This study combined network pharmacology with experimental verification to investigate purslane’s therapeutic mechanism for ulcerative colitis and provide new insights into treatment strategies.

## 2. Results

### 2.1. Identification of Potential Therapeutic Targets of POAs

Following a systematic in silico screening process utilizing the TCMSP, Swiss Target Prediction, and SuperPred databases, a total of 142 potential therapeutic targets associated with POAs were identified. This comprehensive set of targets was obtained after rigorous prediction, organization, and deduplication. To further enhance transparency regarding the identified bioactive compounds, Table 1 now includes a clear classification of alkaloids. Specifically, the original study identified 10 candidate bioactive compounds via ADME screening, of which 2 were definitively classified as alkaloids (isobetanidin and isobetanin_qt), with the remaining 8 belonging to other compound classes (e.g., flavonoids, saponins). The complete list of these 10 compounds, detailing their names, structures, and categories, is fully presented in revised Table 1.

### 2.2. Identification of UC-Related Targets and Overlapping Targets

**Figure 1:**
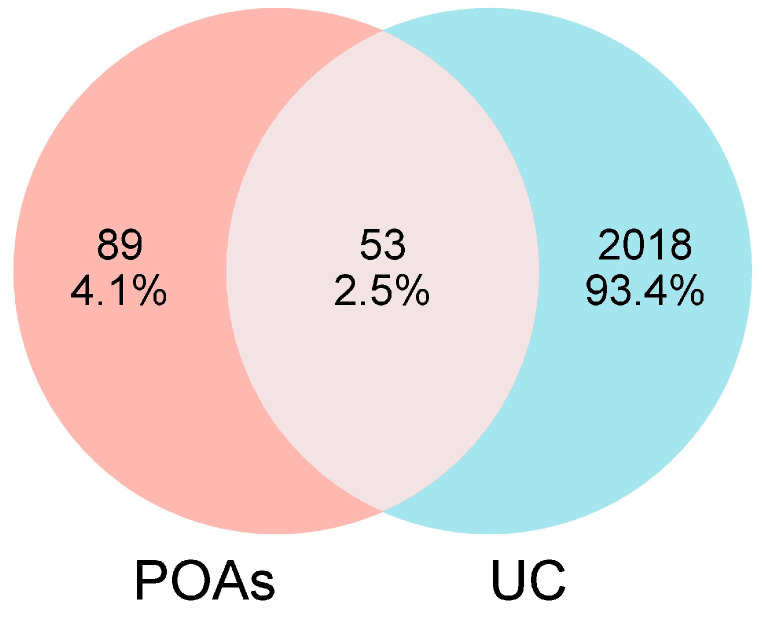
Venn diagram of targets from UC and POAs.

A thorough search and screening of the OMIM and GeneCards databases yielded a total of 2071 targets implicated in UC. Comparative analysis between the identified POA-associated targets and the UC-related targets revealed a significant overlap of 53 common targets. These 53 overlapping targets represent the key molecular nodes through which POAs are hypothesized to exert their therapeutic effects on UC. The detailed list of these common targets is presented in Figure 1.

### 2.3. Network Construction and Analysis of “POAs—Intersection Targets”

**Figure 2:**
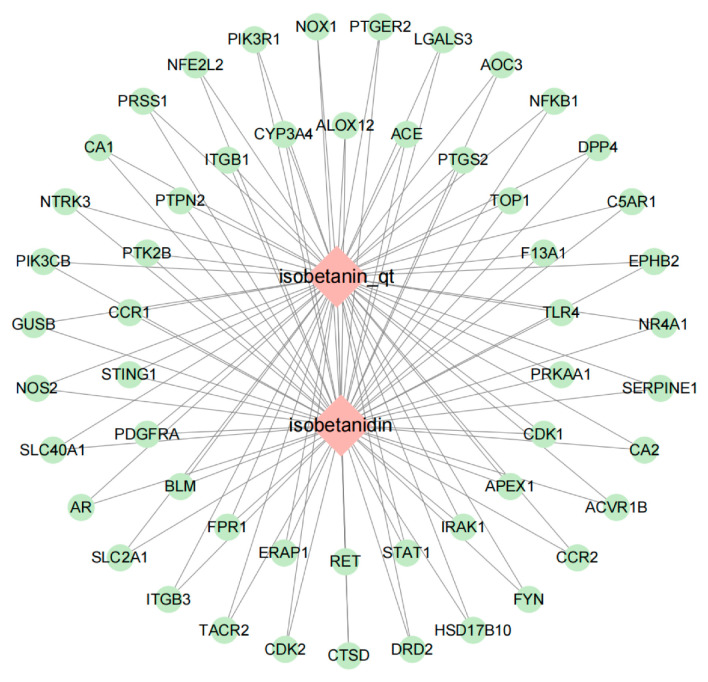
POAs—Intersection targets.

To visualize the molecular interactions within the identified “POA—Intersection Targets” network, Cytoscape version 3.10.0 was employed for network construction. The resulting network comprised a total of 55 nodes and 106 interaction edges. In this network representation: Diamonds denote active compound molecules. Circles represent the intersection targets common to both POAs and UC. Connecting lines illustrate the direct interactions between the active compound molecules and their respective target proteins. The visual representation of this “POAs—Intersection Targets” network is depicted in Figure 2.

### 2.4. Protein–Protein Interaction (PPI) Network Analysis

The PPI network was constructed by importing the TSV file obtained from the STRING database into Cytoscape version 3.10.0. To identify the most crucial protein targets within this network, the CentiScape 2.2 plugin was utilized for topological analysis. Key target nodes were prioritized based on their centrality measures, with the size of the node circle and the intensity of its color indicating higher degree values.

**Figure 3:**
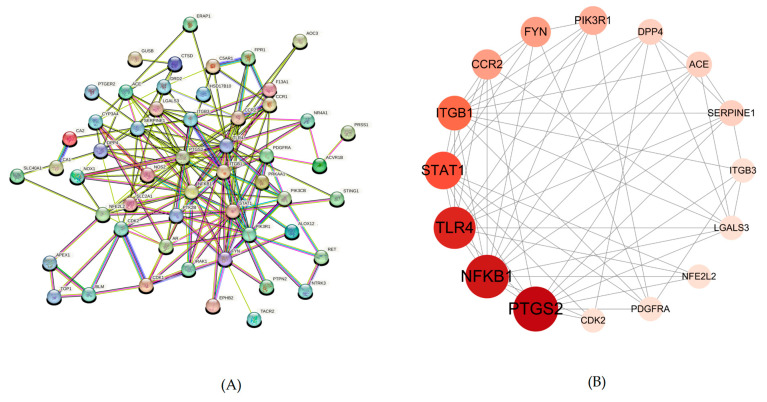
This is a PPI network. (**A**) PPI network of potential targets of POAs in the treatment of UC. (**B**) Topology analysis results of potential targets interaction.

The resulting PPI network, representing the interactions of POAs targets involved in the treatment of UC, comprised 51 nodes and 172 interactions. Following the analysis with CentiScape 2.2, 16 key targets were identified. Among these critical targets, several were highlighted, including PTGS2, NFKB1, TLR4, STAT1, ITGB1, and FYN. The visual representation of this PPI network, highlighting these key targets, is presented in Figure 3.

### 2.5. Gene Ontology (GO) Function and Kyoto Encyclopedia of Genes and Genomes (KEGG) Pathway Enrichment Analysis of Intersection Targets

To elucidate the biological functions and signaling pathways regulated by the identified intersection targets common to POAs and UC, GO functional enrichment and KEGG pathway analyses were performed using the Metascape platform.

**Figure 4:**
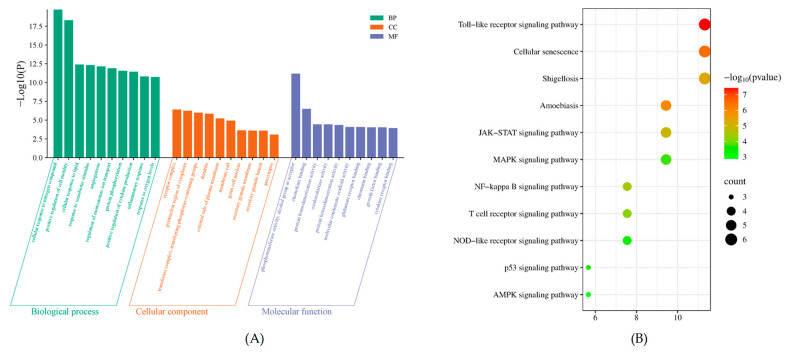
Bioinformatics analysis of target proteins of POAs against UC. (**A**) GO function enrichment of targets of POAs for UC treatment. (**B**) KEGG enrichment of targets of POAs for UC treatment.

The GO enrichment analysis revealed a substantial number of functional annotations, yielding 658 terms for Biological Process (BP), 62 terms for Cellular Component (CC), and 79 terms for Molecular Function (MF). The top 10 significantly enriched terms, ranked by their adjusted *p*-value, are presented in Figure 4A.

KEGG pathway enrichment analysis was conducted on the intersection targets to identify relevant signaling pathways modulated by POAs in the context of UC treatment. A total of 113 functional entries were identified through this analysis. The most significantly enriched KEGG pathways, based on *p*-value screening and visualized via Metascape, included well-established pathways such as the Toll-like receptor (TLR) signaling pathway, the NF-kappa B signaling pathway, and the Mitogen-activated protein kinase (MAPK) signaling pathway. These results indicate that POAs exert their anti-UC effects through multi-target regulation of inflammatory, immune, and metabolic pathways (Figure 4B).

### 2.6. Effect of POAs on Caco-2 Cell Viability

**Figure 5:**
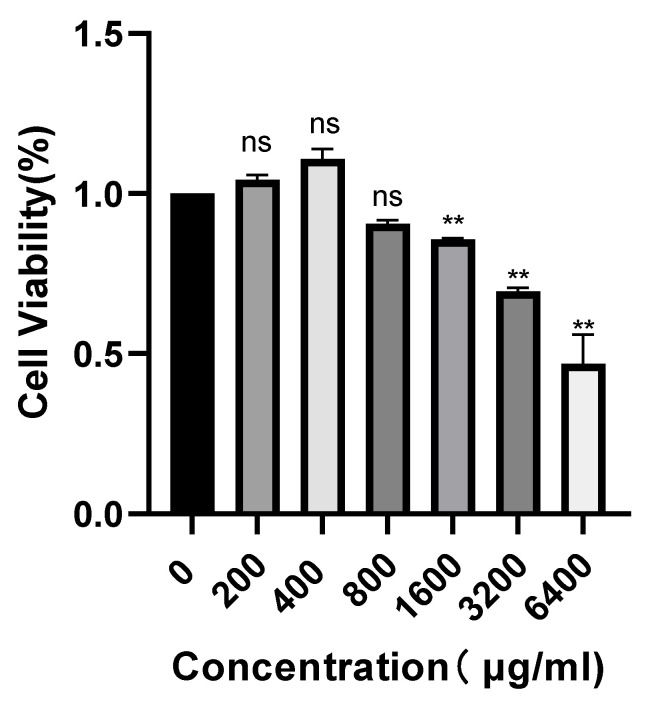
Concentration-dependent effect of POAs on Caco-2 cell viability (“ns” stands for “non-significant”, ** *p* < 0.01 vs. the Control group).

Figure 5 presents the concentration-dependent impact of POAs on Caco-2 cell viability. Under standard culture conditions, POAs treatments at 200 and 400 μg/mL resulted in a non-significant (ns) marginal increase in cell viability relative to the control (0 μg/mL). Significantly, while concentrations up to 800 μg/mL demonstrated no appreciable cytotoxicity, a substantial decrease in cell viability was observed at 1600 μg/mL and higher (*p* < 0.01). These findings delineate a potential therapeutic window for POAs, highlighting their cytotoxic capacity at elevated doses and emphasizing the necessity of carefully selecting dosage parameters for subsequent anti-inflammatory investigations.

### 2.7. Effect of POAs on Caco-2 Cell Migration

**Figure 6:**
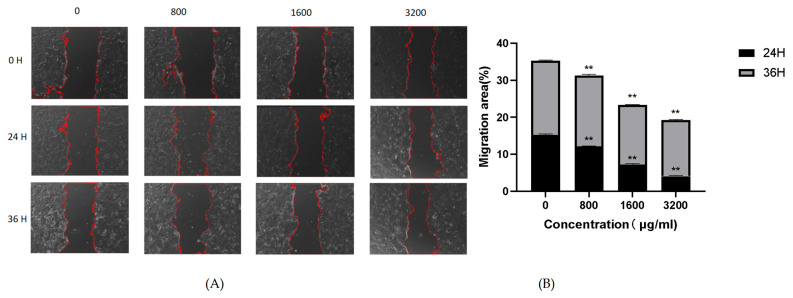
Effect of POAs on Caco-2 cell migration. (**A**) Representative microscopic images of wound healing assay at different time points under POAs treatment. Red dashed lines indicate the wound edges; (**B**) Quantification of cellular migratory area following 24 and 36-hour treatment with POAs. (** *p* < 0.01 vs. the Control group).

The influence of POAs on Caco-2 cell migration was evaluated utilizing a wound healing assay, the results of which are presented in Figure 6. Comparative analysis with the untreated control (0 μg/mL) demonstrated a significant suppression of wound closure in POAs-treated cell cultures. This observed inhibition was clearly contingent upon both the concentration of POAs and the duration of exposure. As depicted in Figure 6A, the extent of wound closure progressively diminished over 24 and 36 h with increasing POAs concentrations (800, 1600, and 3200 μg/mL). The quantitative data in Figure 6B further substantiates this inhibitory trend, revealing a significant reduction in the percentage of wound closure at POAs concentrations of 1600 μg/mL and 3200 μg/mL (**, *p* < 0.01) relative to the control. These findings collectively indicate that POAs possess potent anti-migratory activity against Caco-2 cells, with their efficacy being dose dependent.

### 2.8. Effects of POAs on DAI Scores of UC Mice

**Figure 7:**
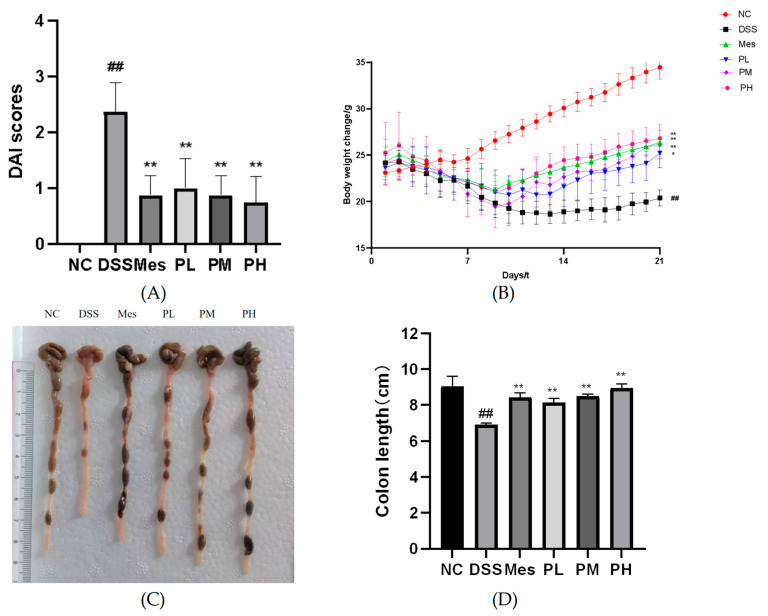
Effects of POAs on the general condition and colonic health of DSS-induced UC mice. (**A**) DAI scores over the experimental period. (**B**) Body weight changes throughout the study. (**C**) Representative macroscopic images of colons from each experimental group. Scale bar indicates 1 cm. (**D**) Colon length at the end of the experimental period. (NC: Control group. DSS: Model group. Mes: Mesalazine group. PL: Low-dose POAs group. PM: Medium-dose POAs group. PH: High-dose POAs group. Figure 8, Figure 9, Figure 10 and Figure 11 are the same (** *p* < 0.01 vs. the Model group; ## *p* < 0.01 vs. the Control group).

Figure 7A demonstrates that mice induced with UC exhibited significantly elevated Disease Activity Index (DAI) scores compared to the healthy blank control group (*p* < 0.01). In contrast, therapeutic intervention with high-dose POAs or combination therapy significantly ameliorated these DAI scores, reducing them to levels comparable to those observed in the blank control group (*p* < 0.01 compared to the model group). This indicates a substantial reduction in disease severity upon POAs treatment.

### 2.9. Effects of POAs on Body Weight in UC Mice

The impact of POAs on body weight of UC mice is illustrated in Figure 7B. Induction of UC led to a significant decrease in body weight in the model group relative to the blank control group (*p* < 0.01). Conversely, therapeutic interventions with mesalazine (positive control), medium-dose POAs, and high-dose POAs significantly counteracted this weight loss, restoring body weight to levels closer to those of the blank control group (*p* < 0.01 compared to the model group). This suggests a beneficial effect of POAs on mitigating UC-induced cachexia.

### 2.10. Effect of POAs on Colon Length in UC Mice

As shown in Figure 7C,D, mice in the UC group had shorter colons than the blank control group (*p* < 0.01). Treatment with mesalazine (positive control) or POAs at various doses significantly attenuated colon shortening (*p* < 0.01). Notably, the high-dose POAs group demonstrated the most prominent restorative effect, indicating the dose-dependent therapeutic benefit of POAs in ameliorating UC-associated colon pathology.

### 2.11. Effect of POAs on the Histopathological Changes in Colon Tissue of UC Mice

**Figure 8:**
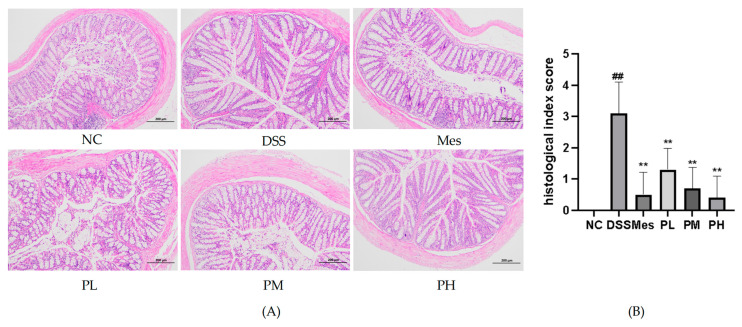
Pathological changes in colon tissue of mice in each group (HE staining, ×100). (**A**) Mouse colon HE staining (a scale bar representing 200 µm was added to the image for scale reference); (**B**) semi-quantitative pathological scoring of colon tissue damage (** *p* < 0.01 vs. the Model group; ## *p* < 0.01 vs. the Control group).

Histopathological examination of colon tissue revealed distinct morphological differences among the experimental groups (Figure 8). The normal control group exhibited intact colonic architecture, characterized by healthy mucosa and submucosa with minimal inflammatory cell infiltration. In stark contrast, the UC model group displayed severe pathological alterations, including significant crypt destruction, loss of goblet cells, submucosal edema, and widespread ulcer formation, indicative of acute inflammation. Treatment with POAs resulted in marked amelioration of these histopathological features. Specifically, both medium- and high-dose POAs groups demonstrated significantly reduced inflammatory cell infiltration, attenuated mucosal edema, and substantial restoration of crypt architecture compared to the UC model group. Notably, the high-dose POAs group showed the most pronounced therapeutic effect, with near-normal colonic morphology, underscoring the dose-dependent benefit of POAs in ameliorating UC-associated colonic tissue damage.

### 2.12. Effect of POAs on Serum Levels of Inflammatory Factors in UC Mice

**Figure 9:**
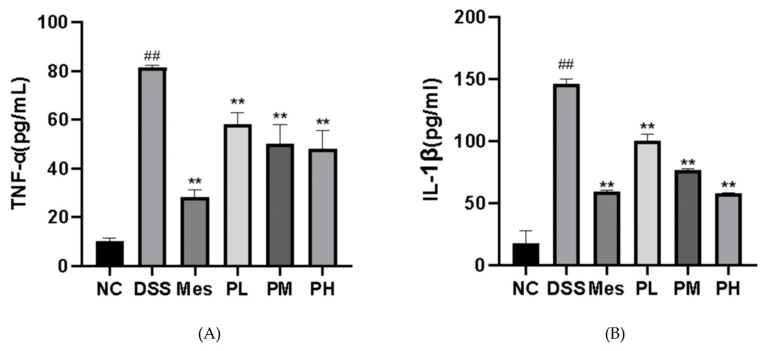
Effects of POAs on serum levels of pro-inflammatory cytokines in DSS-induced UC mice. (**A**) TNF-α serum concentrations; (**B**) IL-1β serum concentrations. Values are presented as mean ± standard deviation (SD) from three independent experiments. (** *p* < 0.01 vs. the Model group; ## *p* < 0.01 vs. the Control group).

As depicted in Figure 9, the serum levels of tumor necrosis factor-alpha (TNF-α) and interleukin-1 beta (IL-1β) in the disease model group were significantly elevated (*p* < 0.01) compared to the blank control group. Conversely, treatment with all tested drugs resulted in a significant reduction in serum TNF-α and IL-1β levels (*p* < 0.01).

### 2.13. Effect of POAs on TLR4/NF-κB Protein Expression in Colon Tissues of UC Mice

**Figure 10:**
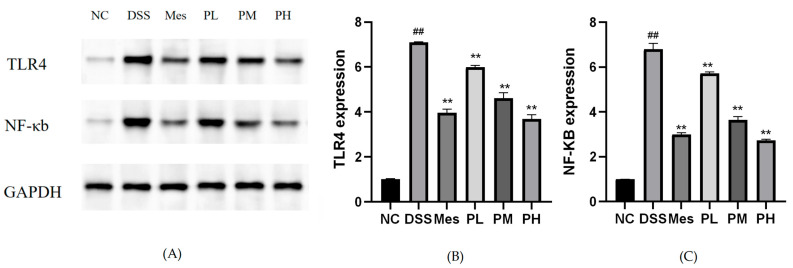
Protein expression levels of TLR4 and NF-κB in intestinal tissues. (**A**) Representative Western blot analysis showing the protein expression levels of TLR4, NF-κB, and GAPDH in different treatment groups; (**B**) Quantitative analysis of TLR4 expression; (**C**) Quantitative analysis of NF-κB expression. Protein levels were normalized to GAPDH. Values are presented as mean ± standard deviation (SD) from three independent experiments. (** *p* < 0.01 vs. the Model group; ## *p* < 0.01 vs. the Control group).

Protein expression levels of TLR4 and nuclear NF-κB were significantly elevated in the colon tissues of the disease model group compared to the blank control group (*p* < 0.01). In contrast, the mesalazine treatment group exhibited significantly reduced TLR4 and NF-κB protein expression (*p* < 0.01). Furthermore, POAs treatment led to a dose-dependent inhibition of TLR4 and NF-κB protein expression (*p* < 0.01; Figure 10).

### 2.14. Effect of POAs on TLR4 and NF-κB mRNA Expression in Colon Tissue of UC Mice

**Figure 11:**
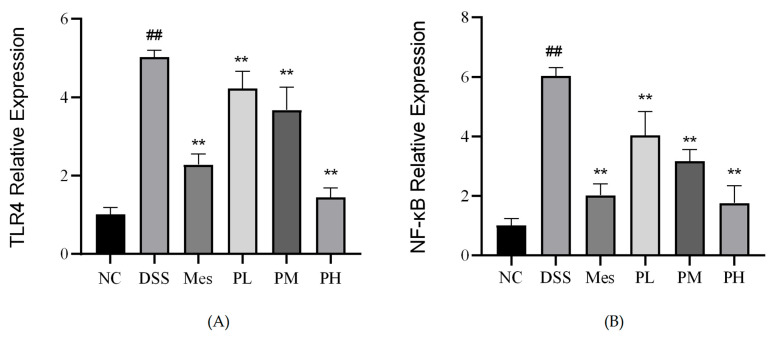
Relative mRNA expression of TLR4 and NF-κB in intestinal tissues. (**A**) Relative mRNA expression of TLR4. (**B**) Relative mRNA expression of NF-κB. Values are presented as mean ± standard deviation (SD) from three independent experiments. (** *p* < 0.01 vs. the Model group; ## *p* < 0.01 vs. the Control group).

Induction of UC in mice led to a significant increase in the mRNA expression levels of TLR4 and NF-κB in colon tissues compared to the control group (*p* < 0.01). As depicted in Figure 11, therapeutic intervention with mesalazine (positive control) and medium-to-high doses of POAs markedly attenuated these UC-induced elevations. Specifically, both medium-dose POAs and high-dose POAs treatments significantly reduced the elevated mRNA levels of TLR4 and NF-κB (*p* < 0.01 vs. model group), restoring them to levels comparable to the control group, although the effect of the medium dose might be slightly less pronounced than the high dose or mesalazine based on visual inspection of typical bar chart trends.

## 3. Discussion

UC, classified in Traditional Chinese Medicine (TCM) as “diarrhea,” “dysentery,” or “intestinal wind,” primarily involves intestinal dysfunction characterized by qi stagnation, damp-heat accumulation, and turbid toxin internalization [13,14]. Modern medicine recognizes UC as a multifactorial disorder involving genetic predisposition, microbial dysbiosis, and immune dysregulation [15]. While conventional therapies (aminosalicylates, corticosteroids, and immunosuppressants) provide symptomatic relief for approximately 50% of patients [16], their limitations necessitate alternative treatments [8].

POL, a medicinal herb documented in the *Chinese Pharmacopoeia*, has demonstrated therapeutic potential in UC management [17]. Ethnopharmacological studies identify multiple bioactive compounds in POL, including flavonoids (apigenin), alkaloids (isobetaine, betalain), and lignans, which exhibit anti-inflammatory and mucosal protective effects [7,18]. POL is described in De Materia Medica as an astringent used to treat headaches, inflammations of the eyes and other organs, heartburn, erysipelas, bladder disorders, tooth numbness, excessive strength, burning sensations, and worms. Numerous clinical trials have reported the safety of POL [19]. Furthermore, polysaccharides from POL ameliorate DSS-induced UC by modulating intestinal homeostasis [20]. Building upon these observations, this study employed network pharmacology to construct a multi-level interaction network of “POAs-target-UC” to unravel its underlying mechanisms.

TLRs are pivotal components of the innate immune system, acting as cell-surface receptors that recognize conserved pathogen-associated molecular patterns (PAMPs), facilitate host–microbe interactions, and orchestrate inflammatory signaling pathways [21]. TLR4, a well-characterized member of the TLR family, recognizes a broad spectrum of PAMPs and directly or indirectly induces the expression of pro-inflammatory factors, thus amplifying inflammatory responses [22]. Both in vitro and in vivo studies, along with clinical investigations, have demonstrated that excessive pathogen exposure in UC triggers heightened TLR4 expression and activation, leading to the upregulation of downstream targets, notably NF-κB [23,24]. NF-κB is a crucial transcription factor involved in diverse cellular biological responses [25], existing primarily as a dimer of p65 and p50 subunits. Following activation, the p65 subunit translocates to the nucleus, binds to the κB sequence of DNA, and initiates the expression of pro-inflammatory mediators, such as IFN-γ, TNF-α, and IL-1β, thereby exacerbating inflammatory damage [26]. It has been reported that traditional Chinese herbs exert anti-inflammatory effects by suppressing the NF-κB/COX-2 signaling pathway activated by lipopolysaccharides [27].

In our in vitro cell-based assays, dose-escalation preliminary experiments with POAs extract, ranging from 200–6400 μg/mL with serial doubling dilutions, revealed that POAs significantly inhibited cell viability (viability < 70%) at concentrations ≥ 1600 μg/mL. In contrast, no overt cytotoxicity was observed within the 800–1600 μg/mL range (viability > 85%). It is common practice in in vitro studies of plant extracts to employ a broad concentration range (e.g., 100–1000 μg/mL) to encompass both pharmacological activity and potential toxicity thresholds [28]. Previous research using Medina longi worm extracts demonstrated low or no cytotoxicity in in vitro toxicity assessments; however, in vivo analyses with high-dose aqueous extracts did exhibit cytotoxic effects, highlighting the dose-dependent nature of plant toxicity [29]. In our in vitro experiments, cells were directly exposed to high concentrations of POAs under static culture conditions. In contrast, in vivo, drug concentrations in target tissues are considerably lower due to metabolism, distribution, and excretion, including first-pass effects in organs like the liver and kidneys.

Subsequently, we established a DSS-induced UC mouse model for in vivo validation. The in vivo results confirmed that POAs not only effectively attenuated systemic inflammation in mice, primarily evidenced by a reduction in serum inflammatory cytokines TNF-α and IL-1β, but more critically, POAs intervention also blocked the transduction of the TLR4/NF-κB signaling pathway. This effectively protected the mouse colon from inflammatory damage. Based on the observed toxicity profile, the experimental condition of ≥1600 μg/mL was strictly defined as an assessment of the cytotoxic threshold and not as a recommended therapeutic concentration. Consequently, we meticulously controlled the POAs dosage in our animal experiments, employing established methods for converting human to animal drug doses as outlined in *Pharmacological Experimental Methods of Traditional Chinese Medicine* [30]. Regarding the correlation between high-concentration toxicity and in vivo therapeutic efficacy, our in vivo treatment involved chronic administration over 14 days. Low-dose, sustained exposure likely exerted therapeutic effects through cumulative modulatory mechanisms, such as anti-inflammatory and antioxidant actions, rather than through acute cytotoxic pathways observed in short-term in vitro high-concentration challenges.

While the TLR4/NF-κB pathway likely plays a predominant role, our network pharmacology analysis suggests that other signaling pathways also warrant investigation for their involvement in the therapeutic effects of POAs against UC. For instance, studies have demonstrated that modulation of the JAK/STAT signaling pathway exerts positive effects in UC treatment [31,32]. Furthermore, microRNA-369-3p has been shown to ameliorate intestinal epithelial inflammation and apoptosis via the MEK/ERK signaling pathway [33] and to modulate intestinal inflammatory responses through the BRCC3/NLRP3 inflammasome axis [33]. Therefore, the precise mechanisms by which POAs exert their effects in UC, and their intricate correlations, necessitate further in-depth validation and exploration in future studies.

## 4. Materials and Methods

### 4.1. Network Pharmacology Analysis

This study employed a network pharmacology approach to elucidate the underlying molecular mechanisms of POAs in managing UC.

#### 4.1.1. Identification of POAs Active Compounds and Potential Targets

Active compounds of POL were retrieved from the Traditional Chinese Medicine Systems Pharmacology Database and Analysis Platform (TCMSP), accessed on 10 May 2024 [34]. To select potentially bioactive candidates, compounds were screened based on oral bioavailability (OB) and drug-likeness (DL), with predefined thresholds of OB ≥ 30% and DL ≥ 0.18. Subsequently, alkaloid components were further filtered, and their corresponding protein targets were identified. The selected alkaloid compounds were processed using the PubChem database [35] (https://pubchem.ncbi.nlm.nih.gov/, accessed on 10 May 2024) to obtain their Simplified Molecular Input Line Entry System (SMILES) strings. These SMILES codes were then submitted to the Swiss Target Prediction platform [36] (http://swisstargetprediction.ch/, accessed on 10 May 2024) and the SuperPred database [37] (http://bioinformatics.charite.de/superpred/, accessed on 10 May 2024) to predict potential protein targets for these active compounds.

#### 4.1.2. Collection of UC-Related Targets

To identify therapeutic targets relevant to UC, a comprehensive search was conducted using the keyword “ulcerative colitis” (or “UC”) within the GeneCards database [38] (https://www.genecards.org/), accessed on 10 May 2024 and the Online Mendelian Inheritance in Man (OMIM) database [39] (https://www.omim.org/, accessed on 10 May 2024). Relevant targets associated with UC pathogenesis and progression were systematically collected.

#### 4.1.3. Identification of Overlapping Targets Between POAs and UC

The intersection of targets identified for POAs and those associated with UC was determined using the Venny 2.1 tool (https://bioinfogp.cnb.csic.es/tools/venny/index.html, accessed on 10 May 2024). Venn diagrams were generated to visually represent the common targets.

#### 4.1.4. Construction of the POAs—Common Target Network

The identified common targets between POAs and UC were imported into Cytoscape version 3.10.0 for the construction of a network. This visualization aimed to delineate the intricate relationships between the active compounds of *Portulaca oleracea* and their shared molecular targets implicated in UC.

#### 4.1.5. Protein–Protein Interaction (PPI) Network Construction and Key Target Identification

The STRING database [40] (https://string-db.org/, accessed on 10 May 2024), a comprehensive resource for functional protein association networks, was utilized to construct a PPI network for the overlapping targets identified for POAs and UC. The analysis was performed for Homo sapiens. The resulting protein interaction data, exported as TSV files, were subsequently imported into Cytoscape version 3.10.0 [41] for further analysis. This process facilitated the identification and visualization of key genes within the overlapping target network.

#### 4.1.6. GO and KEGG Enrichment Analysis

To elucidate the biological functions and pathways associated with the identified common targets, GO functional enrichment analysis and KEGG pathway analysis were performed using the Metascape platform (http://metascape.org, accessed on 11 May 2024) [42]. The enrichment results, illustrating key biological processes and metabolic pathways involved in the therapeutic effects of POAs on UC, were visualized using the bioinformatics online platform (http://www.bioinformatics.com.cn, accessed on 11 May 2024).

### 4.2. Cell Experiments

#### 4.2.1. Materials and Reagents

Human colorectal adenocarcinoma Caco-2 cells (Lot No. CL-0050) and cell-specific culture medium (Lot No. CM-0050) were procured from Wuhan Procell Life Science & Technology Co., Ltd. (Wuhan, China). The total alkaloid fraction of *Portulaca oleracea* L. (POL) was custom extracted under standardized protocols by Jilin City Deer King Pharmaceutical Co., Ltd. (Jilin, China) using ethanol-acid aqueous extraction and subsequent HPLC quality control. The Cell Counting Kit-8 (CCK-8, Lot No. C0005-1 ml) was sourced from Shanghai Tongren Biotechnology Co., Ltd. (Shanghai, China). All cell culture procedures utilized Gibco-supplied reagents, including high-glucose DMEM (Thermo Fisher Scientific, Waltham, MA, USA), fetal bovine serum (FBS), and penicillin–streptomycin (1% *v*/*v*).

#### 4.2.2. Tumor Cell Proliferation Assay (CCK-8)

Caco-2 cells were seeded into 96-well plates (2 × 10^4^ cells/well, 100 µL/well) and incubated for 24 h (37 °C, 5% CO_2_). Cells were treated with six concentrations of POAs extract (0.2, 0.4, 0.8, 1.6, 3.2, and 6.4 mg/mL) for 24 h. After treatment, 10 µL of CCK-8 solution (Shanghai Tongren Biotechnology Co., Ltd., Shanghai, China) was added to each well, followed by 2 h incubation. Absorbance was measured at 450 nm using a microplate reader.

#### 4.2.3. Tumor Migration Wound Healing Assay

Caco-2 cells (2 × 10^5^ cells/well) were seeded in 6-well plates and cultured to 90–100% confluence. A sterile pipette tip was used to create five parallel scratches per well. After washing with PBS (HyClone, Logan, UT, USA), cells were treated with POAs extract. Migration was monitored at 0, 24, and 36 h, with images captured at 10× magnification. Wound closure was quantified using ImageJ software version 1.53t (NIH).

### 4.3. Animal Experiments

#### 4.3.1. Animals 

Forty-eight male ICR mice (specific pathogen-free [SPF] grade, 6–8 weeks old, 18–22 g) were obtained from Changchun Yisi Experimental Animal Technology Co., Ltd. (Jilin, China). Animals were housed under controlled conditions (22 ± 0.5 °C, 55% ± 5% relative humidity, 12 h light/dark cycle) with ad libitum access to standard rodent chow and sterilized water. All procedures were approved by the Institutional Animal Care and Use Committee of Beihua University (Protocol No. 20240703) and complied with ARRIVE guidelines.The ARRIVE guidelines used in this study are provided in Supplementary Materials (SM1).

#### 4.3.2. Reagents and Instruments

POAs was extracted and standardized by Jilin Lvwang Pharmaceutical Co., Ltd. (Changchun, China) using ethanolic-aqueous acid extraction followed by ultraviolet–visible spectrophotometric quantification (total alkaloid content: 21.5 mg/kg dry weight) [43]. Mesalazine (batch no. H20143164) was purchased from Shanghai Aidefa Pharmaceutical Co., Ltd. (Shanghai, China).

Antibodies against TLR4 (batch no. GB11519), GAPDH (batch no. GB15004), and NF-κB (batch no. GB11997) were purchased from Wuhan Servicebio Technology Co., Ltd. (Wuhan, China). Mouse TNF-α and IL-1β ELISA assay kits (Batch Nos. DP05F8D82982 and DP0824D44714, respectively) were purchased from Wuhan Elabscience Biotechnology Co., Ltd. (Wuhan, China).

The Spark™ multifunctional microplate reader (Tecan, Mannedorf, Switzerland), MicroChemi 4.2 gel imaging system (DNR, Jerusalem, Israel), and QuantStudio™ Plus real-time PCR system (Applied Biosystems, Foster City, CA, USA) were used.

#### 4.3.3. Animal Grouping, Modeling, and Drug Administration

**Figure 12:**
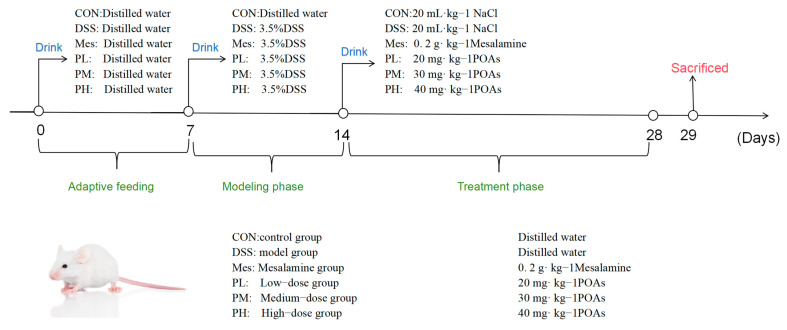
The animal experimental flow. An experimental colitis model was induced by administration of 3.5% DSS in drinking water from the 7th day. POAs were given intragastrically daily for the POAs group from day 14.

The experimental protocol was adapted (Protocol No. 20240703) and optimized from the method described by Yang C et al., based on our preliminary experiments, to establish a murine model of UC [44]. Following a 7-day acclimation period, the mice were randomly divided into six groups (n = 8 per group): (1) Control (CON); (2) Model (MOD); (3) Mesalazine (positive control); (4) Low-dose POA; (5) Medium-dose POA; and (6) High-dose POAs groups. The experiment commenced on Day 0. From Day 0 to Day 7, all groups had free access to distilled water. From Day 7 to Day 14, the MOD, mesalazine, and POAs groups received 3.5% DSS solution in their drinking water to induce colitis. The CON group continued to receive distilled water. The mesalazine group received a mesalazine suspension [45] administered orally at a dose of 0.2 g·kg^−1^ daily. The POAs groups received low-, medium-, and high-dose POAs suspensions, administered orally, at doses of 20, 30, and 40 mg·kg^−1^, respectively. The remaining groups received an equivalent volume of distilled water. The dosage of *Portulaca oleracea* alkaloids was determined based on the crude herb specifications outlined in the *Chinese Pharmacopoeia* [46], considering a human clinical dose of 1–5 g per administration, three times daily (9–15 g total daily). Animal equivalent doses were calculated using the human-to-animal dose conversion methodology described in the *Experimental Methodology of Chinese Herbal Pharmacology* [30]. The entire scheme of this study is shown in Figure 12.

#### 4.3.4. Body Weight and DAI Scoring

The mice were monitored daily for clinical signs of colitis. The Disease Activity Index (DAI) for each mouse was assessed according to the scoring criteria detailed in Table 2. The DAI was calculated as the sum of the weight loss score, diarrhea score, and rectal bleeding score, providing a comprehensive measure of disease severity. The final DAI value for each mouse was determined by the average of these three individual scores, offering an overall indication of the severity of the UC.

#### 4.3.5. Colon Length Measurement

Following ethical guidelines for animal use, the mice were euthanized by cervical dislocation. The entire colon was then carefully excised and immediately rinsed with ice-cold PBS to remove any luminal contents. The colon was then gently straightened and its length was accurately measured in centimeters using a calibrated ruler, providing an objective assessment of colon shortening, a key indicator of inflammation in UC.

#### 4.3.6. Hematoxylin–Eosin (H&E) Staining of Colon Tissues

After euthanasia, the abdominal cavity was opened, and colonic tissues were collected for histopathological analysis and protein expression analysis. The colon sections were immediately fixed in 4% paraformaldehyde. Following fixation, the tissues underwent routine processing: graded ethanol dehydration, paraffin embedding, and sectioning into slices 7 μm thick. Subsequently, the sections were stained with H&E to visualize tissue morphology. Histopathological damage was evaluated by calculating a histological score, with scoring criteria detailed in Table 3. This scoring provided a quantitative assessment of the extent of colonic tissue damage.

#### 4.3.7. ELISA for Serum Inflammatory Cytokines

Serum samples from each experimental group (n = 8/group) were centrifuged at 3000× *g* for 15 min to remove particulate matter prior to analysis. TNF-α (Batch Nos. DP05F8D82982, Wuhan Servicebio Technology Co., Ltd.) and IL-1β (Batch Nos. DP0824D44714, Wuhan Servicebio Technology Co., Ltd.) concentrations were determined using species-specific commercial ELISA kits according to the manufacturer’s instructions. Briefly, 100 μL of 1:2 diluted serum samples were incubated in pre-coated microplates for 120 min at 37 °C. Following three washes with PBS-T (0.05% Tween-20), chromogenic substrates were added and developed for 30 min in darkness. Absorbance measurements at 450 nm were obtained using a spectrophotometric microplate reader. Quadruplicate measurements were performed for each sample, and cytokine concentrations were calculated using four-parameter logistic regression curves generated with recombinant protein standards.

#### 4.3.8. Western Blot Analysis of Protein Expression in Colon Tissues

Colon tissue specimens (n = 8/group) were homogenized in RIPA lysis buffer containing protease inhibitor cocktail (Shanghai Qichun Biological Technology Co., Ltd., Shanghai, China; Batch No. C0001-1 ml) using a Polytron homogenizer (setting 25, 3 × 30 s pulses). Total protein concentrations were determined via bicinchoninic acid (BCA) assay (Batch Nos. E-BC-K318-M, Elabscience Biotechnology Co., Ltd.) with bovine serum albumin standards. Equal amounts (30 μg) of denatured proteins were separated by 10% SDS-PAGE at 100 V for 120 min and transferred to PVDF membranes using a semidry transfer system (25 V, 60 min). Membranes were blocked with 5% non-fat milk in TBST (Tris-buffered saline with 0.1% Tween-20) for 120 min at room temperature before incubation with primary antibodies: mouse anti-TLR4 (1:1000, Batch no. GB11519, Wuhan Servicebio Technology Co., Ltd., Wuhan, China), rabbit anti-NF-κB p65 (1:1000, batch no. GB11997, Wuhan Servicebio Technology Co., Ltd., Wuhan, China), and mouse anti-GAPDH (1:2000, Batch no. GB15004, Wuhan Servicebio Technology Co., Ltd., Wuhan, China) at 4 °C for 16 h. Following three TBST washes (10 min each), membranes were incubated with HRP-conjugated goat anti-mouse/rabbit secondary antibodies (1:5000) for 90 min at room temperature. Protein bands were visualized using enhanced chemiluminescence reagent (Batch no. AR1171, Wuhan Biosharp Biological Engineering Co., Ltd, Wuhan, China) and captured with the ChemiDoc XRS + Imaging System. Band intensity quantification was performed using ImageJ software (v1.53t, NIH) with background subtraction, and target protein expression levels were normalized to GAPDH as loading control.

#### 4.3.9. Quantitative Real-Time PCR (qRT-PCR) Analysis of Colonic Gene Expression

Total RNA was extracted from weighed colon tissue samples using a commercially available RNA extraction kit (Wuhan Servicebio Technology Co., Ltd., Batch no. G3640-50T). The concentration and purity of the extracted RNA were assessed using a NanoDrop spectrophotometer (Thermo Fisher Scientific, Waltham, MA, USA) by measuring the absorbance at 260 nm and 280 nm (A260/A280 ratio).

Reverse transcription of the RNA into complementary DNA (cDNA) was performed using a reverse transcription kit (GenStar Biosolutions Co., Ltd., Beijing, China; Batch no. A230-10) and following the manufacturer’s protocol, using a defined amount of total RNA. RT-PCR was performed using a real-time PCR instrument QuantStudio™ Plus real-time PCR system (Applied Biosystems, USA) with gene-specific primers (Table 4). The primers were designed based on NCBI GenBank (accessed on 10 September 2024) data and synthesized by Sangon Biotech (Shanghai, China). The qRT-PCR reactions were carried out in a total volume of 20 μL containing SYBR Green or TaqMan probe, cDNA template, and the forward and reverse primers. Relative mRNA levels were calculated using the 2^−ΔΔCt^ method, with GAPDH as an internal reference gene for normalization. Data are presented as fold change relative to the control group, expressed as mean ± SD.

#### 4.3.10. Statistical Analysis

All data were analysed using GraphPad Prism 9.0. Normally distributed continuous variables are presented as mean ± SD. Statistical differences between means were determined using independent sample *t*-test and paired sample *t*-test by SPSS 10.0 statistical software. A *p* value ≤ 0.05 was considered statistically significant.

## 5. Conclusions

**Figure 13:**
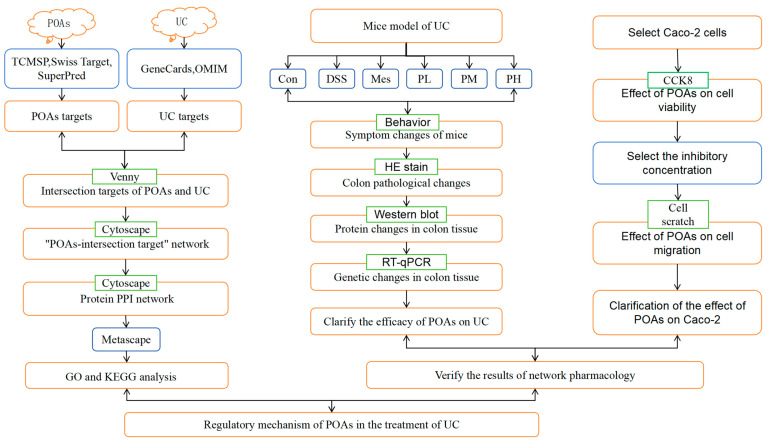
Flowchart of the study.

This study integrated a network pharmacology approach with cellular and animal experiments to investigate the therapeutic potential of POAs for UC (Figure 13). Our findings demonstrated that POAs exert their beneficial effects by modulating the immune-inflammatory response and promoting intestinal mucosal repair. Specifically, the observed effects are likely mediated through a multi-component, multi-target, and multi-pathway synergistic mechanism. These results suggest that POAs represent a promising candidate for the treatment of UC, providing a novel therapeutic strategy and a strong theoretical foundation for future clinical investigations. Further studies are warranted to elucidate the precise molecular mechanisms underlying POAs’ therapeutic actions and to evaluate their efficacy and safety in a clinical setting.

## Figures and Tables

**Table 1:** Basic information of four active ingredients of POL.

Component Name	Mol ID	Molecular Formula	Category
arachidonic acid	MOL001439	C_20_H_32_O_2_	Polyunsaturated fatty acid
cycloartenol	MOL003578	C_30_H_50_O	Triterpenoid
beta-carotene	MOL002773	C_40_H_56_	Carotenoid
beta-sitosterol	MOL000358	C_29_H_50_O	Phytosterol
kaempferol	MOL000422	C_15_H_10_O_6_	Flavonol
5,7-dihydroxy-2-(3-hydroxy-4-methoxyphenyl)chroman-4-one	MOL005100	C_16_H_14_O_6_	Flavonoids
luteolin	MOL000006	C_15_H_10_O_6_	Flavonoids
isobetanidin	MOL006657	C_18_H_16_N_2_O_8_	Betalains
isobetanin_qt	MOL006662	C_24_H_26_N_2_O_13_	Betalains
quercetin	MOL000098	C_15_H_10_O_7_	Flavonoids

**Table 2:** DAI scoring criteria.

Score	Weight Loss/%	Diarrhea	Hematochezia
0	<1	Normal	No bleeding
1	1~5	Loose stools	Occult bleeding
2	6~10	Pasty stools	Visual bleeding
3	>10	Diarrhea	Gross bleeding

**Table 3:** Histological Damage Index scoring criteria.

Score	Epithelial Damage	Inflammatory Infiltration	Crypt Architecture	Ulceration
0	Intact epithelium	No infiltration	Normal crypts	None
1	Focal erosion	Minimal damage	<10% crypt distortion	No ulcers
2	Multifocal erosion	Moderate	10–30% crypt loss	<0.5 mm
3	Extensive erosion	Severe inflammation with microabscesses	30–70% crypt destruction	0.5–2 mm
4	Complete loss	Transmural inflammation with necrosis	>70% crypt loss	>2 mm

**Table 4:** Information for primer sequences.

Gene Name	Primer Sequence (5′→3′)
*TLR4*	F:TCCCTGCATAGAGGTAGTTCC
	R:TCAAGGGGTTGAAGCTCAGA
*Nf-κb*	F:CTCTGGCACAGAAGTTGGGT
	R:TCCCGGAGTTCATCTCATAGT
*GAPDH*	F:GGAGAGTGTTTCCTCGTCCC
	R:ATGAAGGGGTCGTTGATGGC

## Data Availability

The datasets analyzed during the current study are available in the Traditional Chinese Medicine Systems Pharmacology Database and Analysis Platform (TCMSP, https://tcmsp-e.com/tcmsp.php, accessed on 10 May 2024), PubChem database (https://pubchem.ncbi.nlm.nih.gov/, accessed on 10 May 2024), Uniprot database (https://www.uniprot.org/, accessed on 10 May 2024), Gene Cards database (https://www.genecards.org/, accessed on 10 May 2024), Swiss Target Prediction platform (http://swisstargetprediction.ch/, accessed on 10 May 2024), bioinformatics online platform (http://www.bioinformatics.com.cn, accessed on 11 May 2024), Online Mendelian Inheritance in Man (OMIM) database (https://www.omim.org/, accessed on 10 May 2024), and the SuperPred database (http://bioinformatics.charite.de/superpred/, accessed on 10 May 2024).

## Supplementary Materials

The following supporting information can be downloaded at: https://www.mdpi.com/article/10.3390/ijms26146978/s1.
